# Characterization of an Endophytic Strain *Talaromyces assiutensis*, CPEF04 With Evaluation of Production Medium for Extracellular Red Pigments Having Antimicrobial and Anticancer Properties

**DOI:** 10.3389/fmicb.2021.665702

**Published:** 2021-08-04

**Authors:** Rahul Chandra Mishra, Rishu Kalra, Rahul Dilawari, Sunil Kumar Deshmukh, Colin J. Barrow, Mayurika Goel

**Affiliations:** ^1^TERI-Deakin Nano Biotechnology Centre, The Energy and Resources Institute (TERI), TERI GRAM, Gurgaon, India; ^2^Central Council of Scientific Research (CSIR)-Institute of Microbial Technology, Chandigarh, India; ^3^Centre for Chemistry and Biotechnology, School of Life and Environmental Sciences, Deakin University Geelong, Geelong, VIC, Australia

**Keywords:** pigments, endophyte, polyketide, azaphilone, fungal

## Abstract

Considering the worldwide demand for colorants of natural origin, the utilization of ascomycete fungi as a prolific pigment producer unfolds a novel way to obtain these pigments for various applications, including food, cosmetic, and medical use. The presence of very few natural red pigment alternatives in the market also attracts research and industry priorities to unearth novel and sustainable red pigment producers. The present work is an attempt to identify a novel source of red color obtained from endophytic fungi isolated from terrestrial and marine habitats. Based upon the fungal capacity for pigment production, seven isolates of endophytic fungi were recognized as prospective pigment producers. Out of all, fungal isolate CPE04 was selected based upon its capacity to produce profuse extracellular red pigment. The isolate was identified as *Talaromyces assiutensis*, employing morphological features and phylogenetic characterization by internal transcribed spacer (ITS) sequences. To understand the chemical behavior of pigment molecules, an investigation of the chemical profile of fungal culture filtrate dried powder (CFDP) was performed using ultra-high-performance liquid chromatography-diode array detector-mass spectrometry (UPLC–DAD–MS). In total, eight compounds having pigment and pharmaceutical application were tentatively identified using UPLC–DAD–MS. Considering the commercial aspect of the stated work, an effort was also made for standardizing the upscaling of the pigment molecule. Investigations were performed for optimum medium and culturing conditions for maximum pigment production. CFDP was found to have a significant antibacterial activity against the bacterial pathogens *Staphylococcus aureus* (MTCC737), *Vibrio cholerae* (N16961), and methicillin-resistant *S. aureus* (MRSA) (ATCC BAA811). The CFDP showed a minimum inhibitory concentration at 64, 128, and 256 μg/ml against *S. aureus*, MRSA, and *V. cholerae*. A concentration-dependent (50–400 μg/ml) anticancer effect on HeLa cancer line was also observed, having a half-maximal inhibitory concentration (IC_50_) at 300 μg/ml. The antioxidant potential of CFDP has also been proven with the help of an antioxidant assay against 2,2′-azino-bis(3-ethylbenzothiazoline-6-sulfonic acid) radical (IC_50_, 32.01 μg/ml); DNA nicking assay and reactive oxygen species were generated in HeLa cancer line cells. The CFDP was also found to have no cytotoxicity toward HEK 293 T cell line using alamar blue (resazurin), a cell metabolic activity reagent.

## Introduction

Colors have a decisive impact in increasing the esthetic appeal of any product, such as food, textiles, cosmeceutics, or pharmaceuticals (Venil et al., 2014). Synthetic colors are primarily used in various applications for imparting color to various products to date. However, in the last few decades, research shows that the synthetic alternatives are unstable, unsafe as well as expensive for industrial usage in comparison to natural pigments (Sen et al., 2019), thereby driving global attention toward natural colorants and products. The global demand for natural food-based pigments with respect to the food industry is estimated to grow up to $3.2 billion by 2027, giving a leap of 8.4% increase in the compounded annual growth rate between the years 2019 and 2027.

Natural colors from microbes attract industrial significance pertaining to its controlled batch growth conditions, optimal productivity, and final product availability, limiting the seasonal and sample variations. International regulatory agencies like the World Health Organization, European Food Standards Authority, and Food and Drug Administration have laid down strict guidelines and a standard dosage for the application of pigments in food, pharmaceuticals, and cosmeceuticals, owing to their toxicologic, carcinogenic, and health-related hazards (Tuli et al., 2015).

Endophytic fungi are a group of diverse ascomycetous fungi that grow within the plant tissues without causing immediate harmful effects (Hyde and Soytong, 2008; Debbab et al., 2013). These endophytic fungi are known to produce secondary metabolites which can serve as a valuable reservoir of lead molecules with varied industrial applications (Rana et al., 2020). Several filamentous fungi and marine microbes are well-known for their ability to produce water-soluble pigments; nevertheless, low productivity is a major hindrance in their commercial exploitation (Hejazi and Wijffels, 2004; Mishra et al., 2020). They produce invaluable natural products possessing varied ecological functions (Firn and Jones, 2003; Agrawal et al., 2020). Many of these natural products are value-added pigment molecules like carotenoids, melanins, and flavins, serving different purposes in their host. Besides protection against photo-oxidation and environmental stress or as a co-factor in enzyme catalysis, these moieties exhibit distinctive chemical and structural diversity with spectacular hues of colors. Apart from these, these fungi produce several non-carotenoid pigments, such as anthraquinones (Baker and Tatum, 1998), complex polyketides (Butler and Day, 1998), and certain riboflavins. In addition, azaphilones like monascorubin, rubropunctatin, and novel monascusones have also been reported from *Monascus* spp. (Juzlova et al., 1996; Ogasawara et al., 1997).

The implementation of the latest biotechnological tools in harnessing the full potential of various microorganisms is an alternate source for the production of natural colorants (Kalra et al., 2020a,b). The use of monascin-like pigments in food since centuries compels us to exploit ascomycetous fungi for the production of red colorants from fungi (Teng and Feldheim, 2001; Feng et al., 2012; Mishra et al., 2021). Since a decade ago, red color of fungal origin has gained much attention in cosmetics, pharmaceuticals, food as well as textile industries (Dufossé, 2018; Lagashetti et al., 2019). One of the much-explored ecological habitats is marine niches, which are studied for their rich organic milieu and heterotrophic microorganisms (Jones, 2000). The present study was aimed to bioprospect the Alibaug coastal area of the Maharashtra region of the Indian continent, and in total, seven endophytes were isolated from the plant parts of *Avicennia marina*. In this pursuit, the objective of the present work was to screen novel fungal strains producing profuse extracellular pigment and to identify the potential strain based on the morphological, molecular, and extrolite characteristics. The best medium composition was studied based on the carbon, nitrogen as well other chemical constituents for maximum extracellular pigment production. An attempt was also made to identify the chemical composition responsible for providing a pigmented nature to cultured filtrate. In addition, bioactivities like 2,2-azinobis (3-ethylbenzothiazoline-6-sulphonic acid) (ABTS) antioxidant activity, antimicrobial, cytotoxicity as well as apoptotic potential were also analyzed.

## Materials and Methods

### Chemicals and Solvents

Dimethyl sulphoxide (DMSO), ABTS, trolox, alamar blue (resazurin) dye, Eagle’s minimum essential medium, antibiotics, and antimycotic solutions were procured from Sigma-Aldrich (Sigma Aldrich India Pvt. Ltd., Bangalore, India). Potato dextrose agar and broth (PDA and PDB, respectively) were purchased from Himedia, India. Methanol and acetonitrile were purchased from Merck, India. The chemicals and solvents used were of analytical and HPLC grade, respectively. Trypsin-EDTA and fetal bovine serum (FBS) were purchased from GIBCO-Invitrogen.

### Isolation and Cultivation of Pigment-Producing Endophytic Fungi

Endophytic fungi were isolated from healthy roots of *A. marina* obtained from the mangrove forests of Alibaug coastal area (18°39′23.9544″ N, 72°52′47.5248″ E), Maharashtra, India, using the protocols of Kjer et al. (2010). Seven pure cultivable fungal isolates (CPEF 01–07), producing a visible pigment or color, were selected for further processing. For the inoculum, the mycelium was sampled from 8 to 10-day-old culture on PDA plates. The cultivation of fungal isolates was done using a previous method with a slight modification (Kumari et al., 2018). Briefly, three fungal plugs (8–9 mm in diameter) from culture plates were inoculated in 1,000-ml Erlenmeyer flasks, each containing 250 ml of PDB medium. The flasks were incubated at 28°C for 10 days in a static and dark condition. After 10 days of fermentation, all the contents of each flask were collected and filtered through a Whatman filter paper using a Büchner funnel to obtain the culture filtrate, which was further lyophilized for further estimation of extrolites.

### UV–Visible Spectrophotometry and Extracellular Polyketide Metabolite Quantification

The culture filtrate diluted in deionized water was used to examine the absorption profile of the pigments in the wavelength range from 200 to 800 nm using a UV–visible spectrophotometer (UV-1800, Shimadzu Corporation, Tokyo, Japan). In addition, to compare the pigment production from all isolates, the polyketide-based pigment metabolites were quantified by measuring the absorption value at 494 nm (*λ*_max_ of carmine red). Carmine is used as the standard for quantification of pigments, as it is considered as the most stable natural red food colorant, owing to its stability toward heat and light (Lebeau et al., 2017). The polyketide-based pigments were expressed as milligram equivalent (mg eq.) of a commercial red standard pigment (carmine) per liter of culture medium (mg eqv. carmine L^–1^). The red carmine polyketide pigment was selected as it absorbs in the UV range (250–270 nm) and visible range (458–520 nm). The calibration curve of the carmine solution (5–200 mg/L) was measured at 494 nm.

### Identification of Fungal Isolate CPEF04 by Morphological and Molecular Methods

#### Morphological Identification

The CPEF 04 isolate cultured on a PDA plate at 28°C for 5 days was used for observing the colonial morphology. The colony characteristics and microscopic features such as conidia, conidiophores, and size and shape of hyphae were examined using a light microscope (Carl Zeiss) through slide cultures (Photita et al., 2005). The morphological feature of the fungal isolate was also observed using scanning electron microscopy (SEM) (Carl Zeiss, Oberkochen, Germany). For SEM-based studies, the fungal isolate was cultured for 3 days in the same condition as mentioned above. After 3 days, fungal disc was collected and treated with modified Karnovsky’s fixative solution (2.5% paraformaldehyde, 2.5% glutaraldehyde, 0.001 mol/L CaCl_2_, and 0.05 mol/L cacodilate buffer) at pH 7.2. The obtained disc was then again washed with cacodilate buffer thrice for 10 min each and subsequently treated with 1% osmium tetra oxide solution and water for 1 h post-fixation (Silva et al., 2011). Finally, the obtained disc was then treated with sterile MQ deionized water thrice for washing and then treated with acetone (25, 50, 75, 90, and 100%) for 10 min for complete dehydration followed by critical point drying (Emitech K850, Berkshire, United Kingdom). Finally, the sample was assembled on double-sided carbon tape coated with gold–palladium placed on aluminum stubs in a sputter coater (Quorum Technologies SC7620, Berkshire, United Kingdom) and analyzed by SEM at an accelerating voltage of 10 kV.

#### Molecular Identification

For molecular identification, the endophytic fungus was cultured in PDB for 7 days at 28°C in a static condition. The freeze-dried fungal mycelium of CPEF04 was taken for genomic DNA extraction with the cetyl trimethylammonium bromide method (Ausubel et al., 1999). The internal transcribed spacer (ITS) region of rDNA was amplified and sequenced with universal primers ITS4 (5′-TCCTCCGCTTATTGATATGC-3′) and ITS1 (5′-CCGTAGGTGAACCTGCGG-3′). PCR amplification was performed according to the methods of Rajeendran et al. (2017). The amplicons obtained were gel-purified by amicon ultra-columns (Millipore, United States), and 20–40 ng was used for sequencing at Eurofins Laboratory Pvt., Ltd. (Bengaluru, India). The sequences were assembled using FinchTV software^1^, and homologies were determined using BLASTn searches against the NCBI GenBank database with closely related matches (Altschul et al., 1997). MEGA software, version 7.0, was used to construct the sequence alignments and phylogenetic tree (Kumar et al., 2016). Sequence alignment was performed using ClustalW, with default parameters. The unrooted phylogenetic tree was obtained using neighbor joining method (Saitou and Nei, 1987), with p-distance substitution model and 1,000 bootstrapping replicates.

### Screening of Culture Media for Growth and Pigment Production by CPEF04

The fungus was inoculated in a 1,000-ml flask containing 200 ml of seven different media *viz.* PDB, Sabouraud dextrose broth (SDB), malt extract broth (MEB), glucose yeast extract malt broth (GYEM), Czapek yeast extract dox broth (CYDB), yeast extract peptone dextrose broth (YEPD), and defined minimal dextrose broth (DMD) (for chemical composition, see Supplementary Table 1) and was incubated at 28°C for 10 days in a dark and static condition. After 10 days, the culture filtrates were collected after filtering them through a Whatman filter paper and were further lyophilized to obtain dry pigmented concentrates. The CFDP was further analyzed using UV–visible spectrophotometry and HPLC to understand the effect of media composition for pigment production.

### HPLC Analysis

The chemical fingerprint of CFDP obtained from PDB was performed using a Waters HPLC system consisting of a 600 quaternary gradient pump with an online vacuum degasser, a 717 auto-sampler, and 2,996 diode array detectors. The separation of the compounds of interest was achieved using a reverse-phase C_1__8_Luna column (Phenomenex, Lorance, CA, United States, 150 mm × 4.6 mm, 5 μm) and a 10-μl injection volume. An HPLC gradient was applied: A (0.8% orthophosphoric acid in water) and B (acetonitrile). The following gradient was used at a flow rate of 1 ml/min: initial, 100% A in 0–5 min; 100% B in 5–45 min; and 100% A in 45–60 min.

### Ultra-High-Performance Liquid Chromatography–Mass Spectroscopy

Waters ACQUITY H-Class system hyphenated with SYNAPT G2 high-definition mass spectrophotometer instrument (WATERS, India) was used to investigate the pigmented metabolites present in the CFDP of CPEF04. Analysis was performed on, ACQUITY UPLC BEH C18, 2.1 x 50 mm, 1.7 μm column (WATERS, India). The LC system was coupled with a Waters Q-time-of-flight mass spectrophotometer with a Z-spray electrospray ionization (ESI) source with a lock spray probe. The analysis was performed in positive ESI mode and controlled by MassLynx 4.1 Software. An UPLC gradient having eluent A (0.1% formic acid in water) and eluent B (acetonitrile) was performed. The following gradient was used at a flow rate of 0.3 ml/min: initial, 2% B increased gradually to 90% B at 30 min, with a hold time 5 min, and then changed gradually to 2% B at 45 min.

### Total Antioxidant Capacity Assay

Antioxidant capacity assay was measured using ABTS assay performed in accordance with a previously described protocol by Re et al. (1999). ABTS^+^ radical cation was produced by reacting 7 mM ABTS aqueous solution and 2.45 mM potassium persulfate and allowing the mixture to stand at room temperature (25°C) in the dark for 12–16 h. The working solution was diluted with ethanol for an initial absorbance of about 0.70 ± 0.02 at 745 nm. Antioxidant capacity was assessed by mixing different sample concentrations (2.5–50 μg/ml) and standard trolox (1–50 μM) with ABTS^+^ working standard to make a final volume of 1 ml. The absorbance was recorded at 734 nm immediately after 6 min of incubation at room temperature. The half-maximal inhibitory concentration (IC_50_) for the test samples and trolox was calculated by plotting the scavenging capacity against the concentration. The results were expressed as trolox equivalent antioxidant capacity (TEAC), μM trolox/g DW. A comparison of the antioxidant activity of the sample was also done with well-known antioxidant commercial standards which include ascorbic acid (AA) and tert-butylhydroquinone.

TEAC was calculated as follows:

TEAC = IC_50_ of trolox (μg ml^–1^)/IC_50_ of sample (μg ml^–1^).

### Antioxidant Activity Using DNA Nicking Assay

The antioxidant activity of CFDP was also assessed by DNA damage protection assay. An analysis was performed using supercoiled pBSK plasmid DNA according to the method of Zhao et al. (2014) with slight modifications. A mixture of plasmid DNA (0.5 μg) and CFDP in the concentration range of 25–200 μg/ml was incubated at room temperature for 10 min, followed by addition of an equal volume of Fenton’s reagent (30 mM H_2_O_2_, 80 mM FeCl_3_, and 50 mM ascorbic acid). The reaction mixtures were then allowed to be incubated for 30 min at 37°C. The DNA was examined on 1% agarose gel using ethidium bromide staining. Curcumin was found as positive control.

### Antimicrobial Assay

#### Microbial Strains and Culture Media

Two Gram-positive and two Gram-negative anthropogenic bacterial pathogens—*Staphylococcus aureus* (MTCC737), *Salmonella typhimurium* (MTCC 734), *Vibrio cholerae* (N16961), and methicillin-resistant *S. aureus* (MRSA) (ATCC BAA811)—were obtained from the Microbial Type Culture Collection, Institute of Microbial Technology Chandigarh, India. The obtained cultures were propagated on Luria broth agar (LBA) plates.

#### Disc Diffusion Assay

The antibacterial activity of CPEF04 CFDP was evaluated by disc diffusion method. The test organisms were grown in LB broth overnight. On the next day, 0.1% inoculum from the overnight-grown culture was added in fresh LB medium and grown till 3 × 10^–8^ colony-forming units (CFU)/ml (0.5 McFarland standards); 100 μl of this culture was taken and spread over fresh LBA plates using a sterile L-shaped glass spreader. Under aseptic conditions, sterile 6-mm Whatmann paper no. 1 discs were placed on LBA plates. The fungal CFDP dissolved in 10% aqueous DMSO was used to prepare a stock solution of 80,000 μg/ml and serially diluted (40,000, 20,000, and 10,000 μg/ml); 5 μl from each serially diluted tube was used to load on a disc, giving an amount of 400, 200, 100, and 50 μg per disc. Similarly, controls were also loaded on discs and air-dried under aseptic conditions. The antibiotics—like vancomycin in the case of *S. aureus* and MRSA, streptomycin in the case of *S. typhimurium*, and ampicillin in the case of *V. cholera*—served as positive control, whereas the solvent 10% aqueous DMSO served as the negative control. The agar plates were then incubated overnight at 37°C and observed for a clear inhibition zone which was measured in millimeter (mm).

The broth microdilution method was used to determine the MIC as per the method described by Eloff (1998) according to the Clinical and Laboratory Standards Institute (2015) guidelines. The MIC test was performed in 96-well plates using the previously described modified method. A stock solution of CFDP (1 mg/ml) was prepared in a solvent of 10% aqueous DMSO. Serial dilutions from stock solution were made, ranging from 512 to 16 μg/ml using LB broth. Bacterial suspension was diluted in LB broth to reach approximately 3.0 × 10^–8^ CFU/ml from an overnight-grown culture. From this suspension, 100 μl was inoculated into each well. Streptomycin, ampicillin, and vancomycin (50 μg/ml) served as the positive controls, whereas LB with 10% aqueous DMSO served as the negative control. LB with bacterial inoculums only was also taken as blank. The plates were then incubated overnight at 37°C. After that, 30 μl of alamar blue (0.015%) (resazurin) was added to all wells and incubated for 2 h. The color change from purple to pink indicates bacterial growth. The MIC value was taken as the lowest concentration of crude pigment extract that inhibits the growth of bacteria. Fluorescence was measured at excitation 530 nm and emission at 590 nm (Elshikh et al., 2016).

### Cell-Based Assay

#### Cell Culture

HeLa cells (ATCC CRM-CCL-2) were used for cellular experiments for cell death, cell cycle, cell death type, and ROS generation detection. The plasticwares used were of tissue culture grade and procured from BD Falcon^TM^. All the chemicals used were of analytical grade. Cell culture media were supplemented with 10% (v/v) FBS, 100 U/ml penicillin G, 100 mg/ml streptomycin, and 1 × antimycotic solution. The cell line was maintained at 37°C in a humidified atmosphere containing 5% CO_2_ in a CO_2_ incubator.

#### Cytotoxicity Assay

The cytotoxicity for CPEF 04 CFDP was assessed toward HEK 293T cell line using alamar blue (resazurin), a cell metabolic activity reagent (Sigma-Aldrich). The log phase cells were harvested, and cell count was adjusted to 1 × 10^4^/well in DMEM containing 10% FBS. These were incubated for 12 h under 5% CO_2_ at 37°C in 96-well microplates. On the next day, the cells were incubated with varying concentrations of CFDP at 37°C for 24 h. Following this, 0.02% alamar blue reagent was added, and the cells were further incubated for 6–8 h under 5% CO_2_ at 37°C. Fluorescence was measured with excitation wavelength at 545 nm and emission wavelength at 590 nm in a BioTek plate reader. The percent difference between treated and untreated cells (% viability) was calculated by the following formula:

% viability = (relative fluorescence unit, RFU, of the treated sample/RFU of the untreated cells) × 100.

### Live Dead Cell Assay Using 7-AAD Staining

The effects of CPEF04 CFDP on HeLa cells were studied using 7-aminoactinomycin D (7-AAD) staining as per the standard protocol. Briefly, 10,000 cells were plated in 24-well Tc-grade plates. The cells were incubated overnight at 37°C in a 5% CO_2_ incubator for adherence. On the next day, the cells were incubated with different concentrations of CPEF04 (50, 100, 200, 300, and 400 μg/ml) for the next 48 h. The cells were harvested using trypsin-EDTA formulation and washed with serum-free media. The cells were stained with 1 μg/ml 7-AAD before acquisition in FACS BD Accuri^TM^. The untreated cells were used as the control. Data was plotted in GraphPad prism software, and significance was calculated using unpaired *t*-test.

### Measurement of Intracellular Reactive Oxygen Species

Reactive oxygen species (ROS) generation in CPEF04-mediated apoptosis was checked using fluorescent dye 2′,7′-dichlorodihydrofluorescein diacetate (DCFH-DA) (Sigma Aldrich) in accordance with the method given by Kumari et al. (2017). A total of 10,000 HeLa cells were plated in 24-well plates and rested for 24 h at standard tissue culture conditions. The cells were treated with CPEF04 at 50, 100, 200, 300, and 400 μg/ml for 48 h. The cells were harvested, washed with phosphate-buffered saline, and stained with dye at 50-μM concentration for 15 min in the dark at 37°C. Then, 800 μM of H_2_O_2_ was used as positive control, and untreated cells were used as negative control. The fluorescence generated by dye correlates with an amount of ROS generated using FACS BD Accuri^TM^.

### Statistical Analysis

All the experiments were performed in triplicate, and quantitative variables are represented in terms of mean ± SD in histograms. For statistical significance, the means ± SD of all groups were compared. A total of 10,000 cells were acquired for FACS per tube. For statistical significance, the means ± SEM of all groups were compared. A probability of *P* ≤ 0.05 was taken to indicate statistical significance.

## Results and Discussion

### Isolation of Endophytic Fungi

**FIGURE 1:**
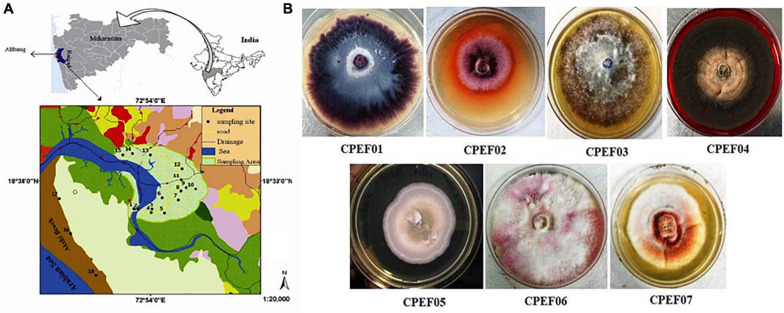
**(A)** Map of a mangrove forest area, Alibaug, Maharashtra. **(B)** Fungal isolates obtained from the roots of the mangrove plant *Avicennia marina*.

A total of seven different cultivable color-producing endophytic fungi were selected from the roots of the marine mangrove plant *A. marina* collected from the mangrove forest of Alibaug coastal area, Maharashtra (Figure 1A), using point inoculation technique for obtaining pure culture using the method mentioned above. Subsequently, the isolates were sub-cultured on PDA medium (Figure 1B) for propagation of pure culture. Out of these seven, five strains were found with diffusible pigments on PDA plates. The pure fungal isolates were propagated using PDB medium, and 8–10-day-old cultures were used for further study.

### Selection of Elite Strain for Extracellular Pigments

**FIGURE 2:**
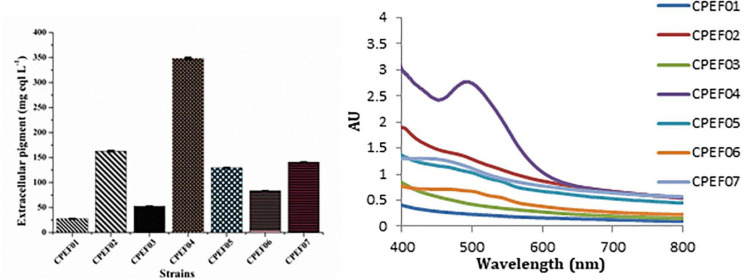
UV–visible absorption spectra of seven different isolates and a comparison of the extracellular pigments produced (mg eqv. carmine L^–1^) by these isolates.

Out of seven pigments producing isolates, elite isolate (CPEF04) was selected based upon maximum extracellular pigment production analyzed with the help of UV–visible spectrophotometry. The absorption spectra of all these strains displayed *λ*_max_ in visible range (400–800 nm); however, maximum absorption was found in strain CPEF04 (Figure 2). The maximum absorbance at 494 nm in CPEF04 further confirms the presence of a red color range, which is equivalent to a commercial red pigment, i.e., carmine. Therefore, estimation of extracellular polyketide-based pigment production by these fungal strains was represented as milligram equivalents of carmine per liter of culture filtrate (Supplementary Figure 1). Table 1 depicts the estimation of milligram equivalents of carmine per liter in culture filtrate. The results showed that lowest extracellular polyketides were observed in isolate CPEF01; on the other hand, isolate CPEF04 produces the maximum extracellular polyketide-based pigment which is 348.1 ± 2.28 mg equivalents carmine L^–1^ (Figure 2).

**TABLE 1:** Extracellular pigment production by seven endophytic fungi isolated from the roots of *Avicennia marina*.

Fungal strain	Extracellular pigments
	(mg eqv. carmine L^–1^)^a^
CPEF01	27.46 ± 0.70
CPEF02	162.92 ± 1.38
CPEF03	52.53 ± 0.64
CPEF04	348.17 ± 2.28
CPEF05	129.57 ± 0.59
CPEF06	83.44 ± 0.19
CPEF07	140.63 ± 0.66

*^*a*^mg eqv. carmine L^–1^ ± standard deviation (*n* = 3).*

### Morphological Identification and Molecular Characterization of Endophytic Isolate CPEF04 Obtained From *A. marina*

**FIGURE 3:**
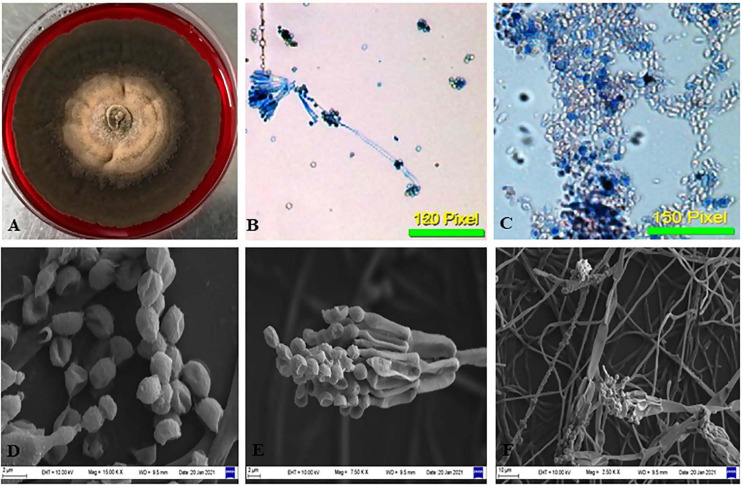
Morphological examination of the isolate CPEF04 from *Avicennia marina*. **(A)** Obverse of colonies of CPEF04. **(B,C)** Light micrographs of the conidiophores and conidia of isolate CPEF04. **(D–F)** SEM of the structures of the strain CPEF04. **(D)** Conidia and **(E)** structure of the conidiophore. **(F)** Surface of the colonies.

The isolate CPEF04, selected on the basis of UV–visible spectrophotometry for producing profuse extracellular pigments, was identified using morphological and molecular characteristics. Colonies reaching 10–12 cm in diameter after 5 days on PDA plates were found to be radially flat, lanose, and scalloped edge. The color was found to be variable with respect to sporulation state, ranging from grayish–olive to dark brownish. The reverse of the plate was dark red. The odor of the culture was musty. The culture produces a diffusible red pigment observed in the media (Figure 3A). Based upon the above-mentioned characteristics, the isolate CPEF04 was found to be a similar species of *Talaromyces* (Figures 3B–F). Furthermore, its ability to exude complicated red pigments into the media highlights its resemblance with marine isolates. Studies on *Talaromyces* sp. pigments have attracted ample attention from pigment industries as they lack toxins and secrete pigments ranging in hues from yellow to red to dark violet (Thrane et al., 2017). Recent inferences have demonstrated that one of the *Talaromyces* strains, *Talaromyces amestolkiae*, produces more extracellular pigments than the intracellular pigment in submerged conditions (de Oliveira et al., 2021).

**FIGURE 4:**
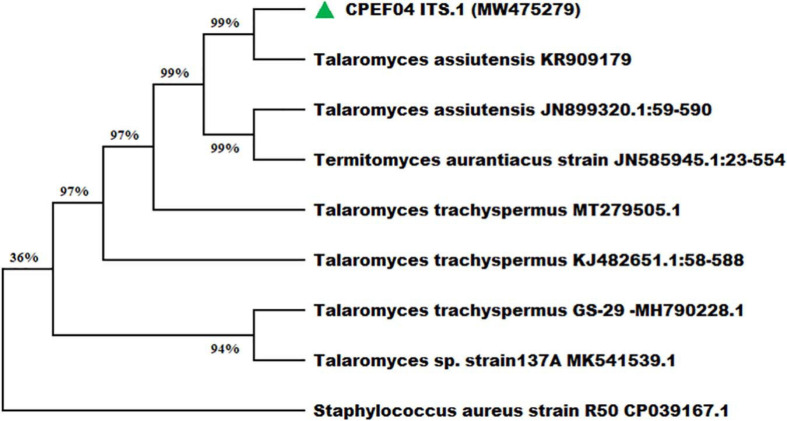
Phylogenetic relationship of endophytic isolate CPEF04 based on ITS1, 5.8S rDNA, and ITS2 regions of DNA sequences. The maximum composite likelihood substitution model was used for phylogenetic reconstruction. The numbers on the tree branches indicate the percentage of bootstrap replications derived from 1,000 replications.

Nevertheless, the morphology-based identification was further supported by molecular-based identification which further provides the taxonomic details of species. The studies based upon amplification using ITS 1 and 4 primers provided a 528-bp-sized gene product, which was further sequenced and deposited to GenBank, with accession number MW475279. The relationship between isolate CPEF04 and its close relatives was studied using a subsequent homology analysis through BLASTn which revealed maximum (99%) similarity with *Talaromyces assiutensis* (GenBank accession number KR909179). Furthermore, the phylogenetic tree for the CPEF04 isolate was constructed using Mega X software as depicted in Figure 4. A further categorization of this isolate into the genus *Talaromyces* confirmed that it belongs to the order *Eurotiales* in the phylum *Ascomyta.*

### Optimization of Culture Media for Growth and Extrolite Pigment Production by CPEF04

**FIGURE 5:**
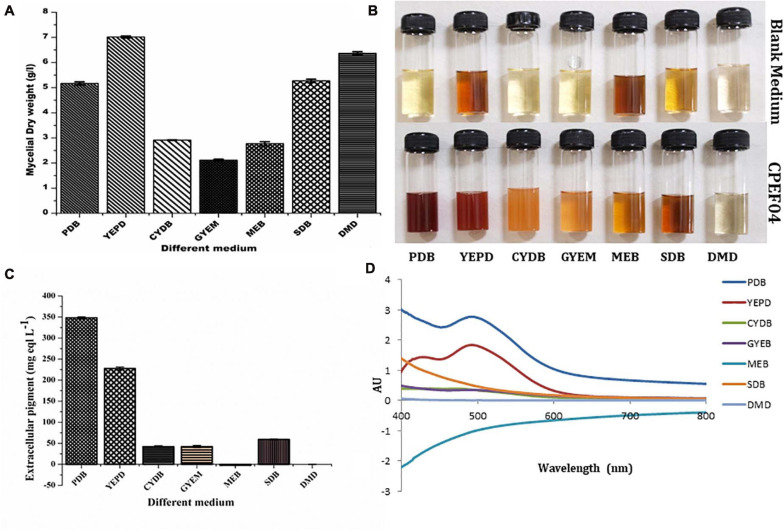
Effect of different media composition on the biomass **(A)** and extracellular pigment production **(B,C)** by CPEF04 isolates. Effect on UV–visible absorption spectra of mycelium filtrate cultured on different media **(D)**.

Seven different growth media were used to find the appropriate medium for optimum growth and extrolite pigment production by the endophytic fungal isolate CPEF04. The production of biomass from different media varied between 2.77 ± 0.08 and 7.01 ± 0.04 g/L (Figure 5A; Supplementary Table 2). The maximum biomass was obtained from YEPD medium following DMD, SDB, PDB, CYDB, MEB, and GYEM. On the other hand, profuse extrolite red pigment was obtained from PDB, followed by YEPD, SDB, GYEM, CYDB, MEB, and DMD (Figures 5B–D). Our finding suggests that a complex source of carbon such as potato starch in PDB favors pigment production, which is also reported by a number of other studies (Lebeau et al., 2017; Thiyam et al., 2020). On the other hand, a complex source of nitrogen such as yeast extract and tryptone in YEPD and SDB, respectively, favors biomass production together with pigment production. These results are also in agreement with previous reports where the effect of nitrogen on pigment yield and profile has been discussed (Morales-Oyervides et al., 2020). DMD, which is a simple form of carbon and nitrogen, only favors biomass production with no pigment production.

### HPLC Analysis

**FIGURE 6:**
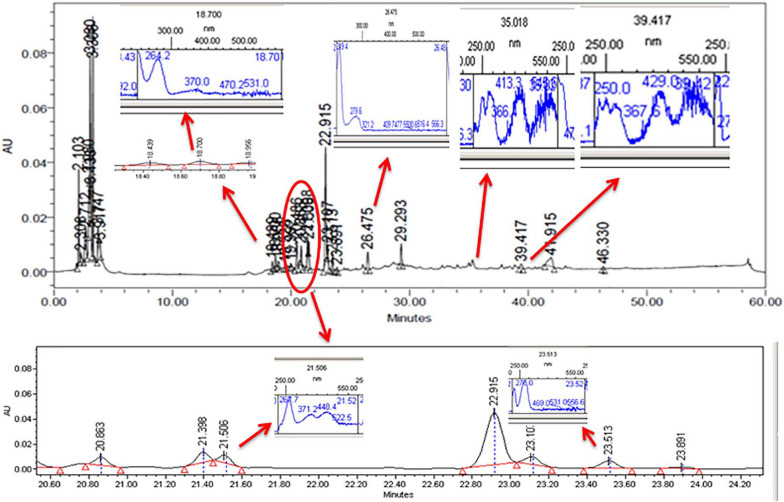
High-performance liquid chromatography–diode array detector chromatogram of the CPEF04 mycelium filtrate. The presence of UV–visible having *λ*_max_ absorption in the range of 412–430 and 514–546 further suggests the presence of *Monascus*-like azaphilone pigments.

The CFDP of CPEF04 was analyzed with the help of HPLC–DAD to understand the nature of molecules by means of the UV–visible absorption spectra of molecules. The UV–vis absorption spectra of peak present at room temperature 18.7, 21.5, 23.5, 26.4, 35.0, and 39.4 suggest the presence of pigment molecules (Figure 6). Peaks present at 35.0 and 39.4, which are showing *λ*_max_ absorption in the range of 412–430 and 514–546, further suggest the presence of *Monascus*-like azaphilone pigments. To further confirm the presence of pigment molecules, CFDP was analyzed with the help of LC–MS.

### UPLC–MS Profiling of the Compounds Present in CPEF04 Isolate

**FIGURE 7:**
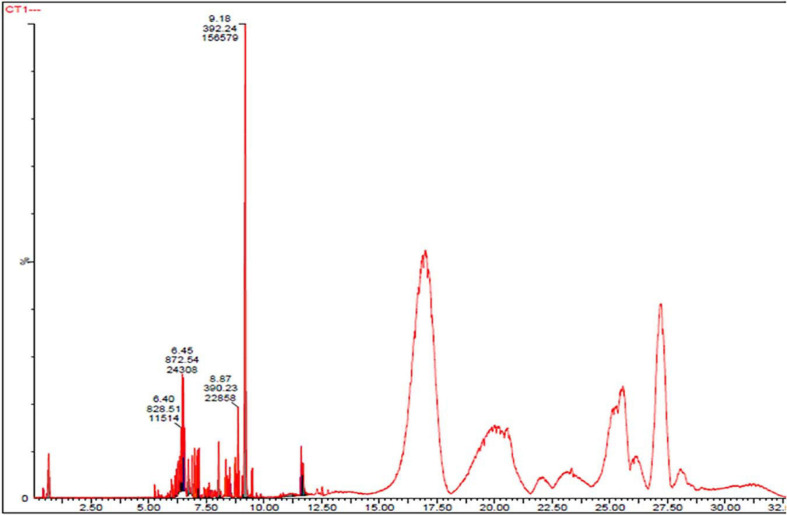
Ultra-high-performance liquid chromatography chromatograms of CPEF04 culture filtrate.

**FIGURE 8:**
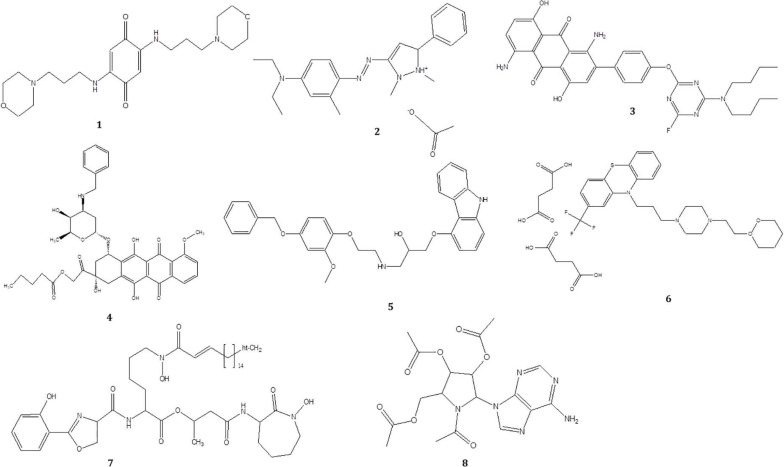
Structure of the compounds tentatively detected on the basis of ultra-high-performance liquid chromatography–mass spectrometry.

To investigate the pigmented molecular composition of fungal CFDP, it has been subjected to LC–ESI–MS. Figure 7 shows the total ion current chromatograms of CFDP of CPEF04. Table 2 presents a list of the few compounds tentatively identified on the basis of their m/z of molecular ion [M+H] ^+^ and respective mass information extracted through their MS and MS/MS spectra, and Figure 8 presents their corresponding structures. A quinone group of compounds which is considered as an essential scaffold for the manufacture of dyes was tentatively identified. The LC of the CFDP showed a peak at retention time 9.18, having m/z 393.2469 corresponding to molecular formula C_2__0_H_3__2_N_4_O_4_ which was identified as p-benzoquinone, 2,5-bis((3-morpholinopropyl)amino). Recent studies have proved these quininone moieties isolated from *Fusarium* sp. to be strong pigment scaffolds with potent bioactivities (Zheng et al., 2017). Similarly, an anthraquinone molecule, which is a widely accepted orange–red pigment also isolated as potent colorants from marine endophytes (Fouillaud et al., 2016), has been tentatively identified. Peak present at retention time 8.34 having m/z 587.2396 corresponding to molecular formula C_3__1_H_3__1_FN_6_O_5_ was identified as 1,5-diamino-2-(4-((4-(dibutylamino)-6-fluoro-1,3,5-triazin-2-yl)oxy)phenyl)-4,8-dihydroxyanthraquinone. Besides these pigment molecules, CFDP was also found to have several bioactive molecules. Peak present at retention time 8.03 having m/z 718.2797 corresponding to molecular formula C_3__9_H_4__3_NO_12_ was identified as N-benzyladriamycin-14-valerate, which is a well-known marketed anticancer and pharmaceutical drug, originally produced by pigment-producing bacteria *Streptomyces peucetius* (Wang et al., 2018). Similarly, peak present at retention time 6.50 having m/z 828.5137 corresponding to molecular formula C_4__4_H_6__9_N_5_O_10_ was identified as mycobactin S, a well-known sideropore and cell transporter already known for its antibacterial activity against *Mycobacterim tuberculosis* (Hu and Marvin, 1997). The presence of such molecules in pigmented CFDP further enhances the pharmaceutical potential and applicability of the dried powder.

**TABLE 2:** Determination of bioactive extrolites in the culture filtrate dried powder (CFDP) of *Talaromyces* sp. CPEF04 by liquid chromatography–electrospray ionization–mass spectrometry (positive ion mode).

Analyte no.	Tentative allotment of compounds based on METLIN	Chemical formula	Retention time	Parent ion (m/z), positive ion mode [M+H]	Peak intensity	Mass recorded (METLIN)	METLIN ID	References
1	p-Benzoquinone,2,5-bis((3-morpholinopropyl)amino)	C_2__0_H_3__2_N_4_O_4_	9.181	393.2469	1.14e7	392.2424	403040	Zheng et al., 2017
2	3-((4-(Diethylamino)-o-tolyl)azo)-1,2-dimethyl-5-phenyl-1H-pyrazolium acetate	C_2__4_H_3__1_N_5_O_2_	8.861	422.2543	9.45e5	421.2478	520966	–
3	1,5-Diamino-2-(4-((4-(dibutylamino)-6-fluoro-1,3,5-triazin-2-yl)oxy)phenyl)-4,8-dihydroxyanthraquinone	C_3__1_H_3__1_FN_6_O_5_	8.344	587.2396	6.97e5	586.234	521177	Fouillaud et al., 2016
4	N-Benzyladriamycin-14-valerate	C_3__9_H_4__3_NO_12_	8.034	718.2797	1.44e6	717.2785	524172	Wang et al., 2018
5	4′-Benzyloxy carvedilol	C_3__1_H_3__2_N_2_O_5_	7.177	513.2385	1.42e6	512.2311	287629	–
6	Oxaflumazine disuccinate	C_3__4_H_4__4_F_3_N_3_O_1__0_S	6.897	744.2799	1.35e6	743.27	389643	–
7	Mycobactin S	C_4__4_H_6__9_N_5_O_10_	6.505	828.5137	2.83e6	827.5044	69367	Hu and Marvin, 1997
8	1-Acetyl-2-[(acetyloxy)methyl]-5-(6-amino-9h-purin-9-yl)pyrrolidine-3,4-diyl diacetate	C_1__8_H_2__2_N_6_O_7_	0.666	435.1627	1.78e6	434.155	546644	–

*–, compound reference was not found.*

### Antioxidant Activity of CPEF04 Isolate Using ABTS Assay

The antioxidant activity was indicated using IC_50_ (concentration of the sample/extract used up to scavenge 50% of the initial ABTS radicals) and TEAC. The lower the IC_50_ and the higher the TEAC value, the greater is the antioxidant activity.

**FIGURE 9:**
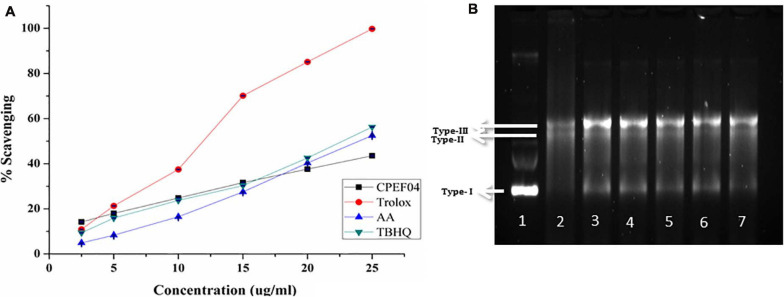
**(A)** Antioxidant activity of the culture filtrate dried powder (CFDP) of isolate CPEF04 and standards (trolox, ascorbic acid, and tert-butylhydroquinone) against the 2,2′-azino-bis(3-ethylbenzothiazoline-6-sulfonic acid radical). **(B)** DNA nicking assay showing the protective effect of CPEF04 against the hydroxyl radical generated by Fenton’s reagent. Lane 1: native plasmid DNA pBSK not treated with Fenton’s reagent; lane 2: DNA treated with Fenton’s reagent; lane 3: DNA treated with Fenton’s reactant and 25 μg/ml curcumin; lane 4–7: DNA treated with Fenton’s reactant and CPEF04 CFDP (200, 100, 50, and 25 μg/ml, respectively).

The radical scavenging activity of culture broth from the fungal isolate CPEF04 against ABTS^+^ radical is illustrated in Figure 9A. The IC_50_ value revealed a significant antioxidant activity, with a TEAC value at 0.25 μM trolox/μg of lyophilized culture filtrate, while an IC_50_ 32.01 μg/ml against ABTS^+^ was obtained. Our findings are in line with the recent activity exhibited by a similar study on *Talaromyces purpureogenus* KKP strain where an IC_50_ of ∼40 μg/ml is reported against ABTS radical (Keekan et al., 2020).

### DNA Nick Assay

Lastly, a DNA nicking assay was used to understand the protective effect of CFDP against hydroxyl radical’s destructive effects on DNA. Hydroxyl radicals generated using Fenton’s reaction mixture resulted in the degradation of the supercoiled form of plasmid DNA (lane 1, type 1) into single-stranded nicked and double-stranded linear forms of DNA (lane 2, types II and III) (Figure 9B). The results showed that the presence of curcumin in 25-μg/ml concentration (lane 3) protects the DNA in supercoiled form. Similarly, treatment with CPEF04 CFDP at different concentrations (200–25 μg/ml; lanes 4–7, respectively) in the reaction mixture diminished the DNA damage. Thus, concentration-dependent intensification of native supercoiled DNA (type 1) was observed.

### Antimicrobial Activity of CPEF04 CFDP

**FIGURE 10:**
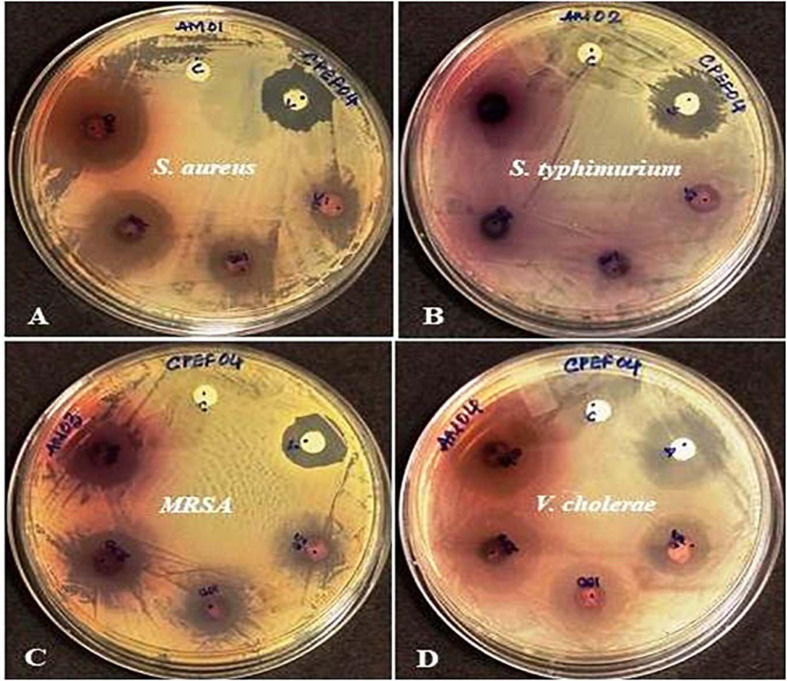
Zones of inhibition produced by CPEF04 culture filtrate dried powder (CFDP) against Gram positive and Gram-negative pathogenic bacteria: **(A)** *Staphylococcus aureus*, **(B)** *Salmonella typhimurium*, **(C)** methicillin-resistant *S. aureus*, and **(D)** *Vibrio cholerae*.

The antibacterial activity of the CPEF04 CFDP was evaluated at different concentrations following the single disc diffusion method (Bauer, 1966). Notable zones of inhibition were observed against *S. aureus*, methicillin-resistant *S. aureus*, and *V. cholera.* No visible zone of inhibition was observed against *S. typhimurium* (Figure 10). The maximum diameter of zone of inhibition was observed in the case of *S. aureus*, MRSA, and *V. cholera* at 400 μg in comparison to the positive control (Table 3). The CFDP was also subjected to MIC determination as per the broth dilution method. The CFDP showed lowest MIC values at 64.0, 128.0, and 256.0 μg/ml against *S. aureus*, MRSA, and *V. cholera*, respectively, which were in congruence with a recent study done on a similar pigment-producing *Talaromyces* strain (Pandit et al., 2018). The findings of our study were in agreement with a similar work by Arivudainambi et al. (2014), in which the ethyl acetate extract of *Alternaria alternata* VN3 isolate was found effective against pathogenic bacteria, with MIC values ranging from 100 to 900 μg/ml.

**TABLE 3:** Antibacterial activity of CPEF04 culture filtrate dried powder (CFDP) [minimum inhibitory concentration (MIC) and zone of inhibition].

	Diameter of zone of inhibition (mm)^a^ against pathogenic bacteria			
	*S. aureus* ^b^	*S. typhimurium*	Methicillin-resistant *S. aureus*^c^	*V. cholerae* ^d^
**Concentrations of CFDP (μg/disc)**				
DMSO (control)	0	0	0	0
50	10.3 ± 0.3	0	10.5 ± 0.3	16.48 ± 0.4
100	16.6 ± 0.3	0	14.14 ± 0.18	20.25 ± 0.2
200	19.3 ± 0.4	0	18.01 ± 0.12	22.21 ± 0.24
400	24.5 ± 0.4	0	22.05 ± 0.07	24.78 ± 0.2
Vancomycin (50 μg/disc)	16.4 ± 0.5	–	14.33 ± 0.11	–
Streptomycin (50 μg/disc)	–	16.4 ± 0.4	–	–
Ampicillin (50 μg/disc)	–	–	–	24.33 ± 0.3

*^*a*^Mean diameter on zone of inhibition ± standard deviation (*n* = 3).*

*^*b*^MIC (μg/ml), 64.0.*

*^*c*^MIC (μg/ml), 128.0.*

*^*d*^MIC (μg/ml), 256.0.*

*–, not applied.*

### Cell Cytotoxicity/Viability Assay

**FIGURE 11:**
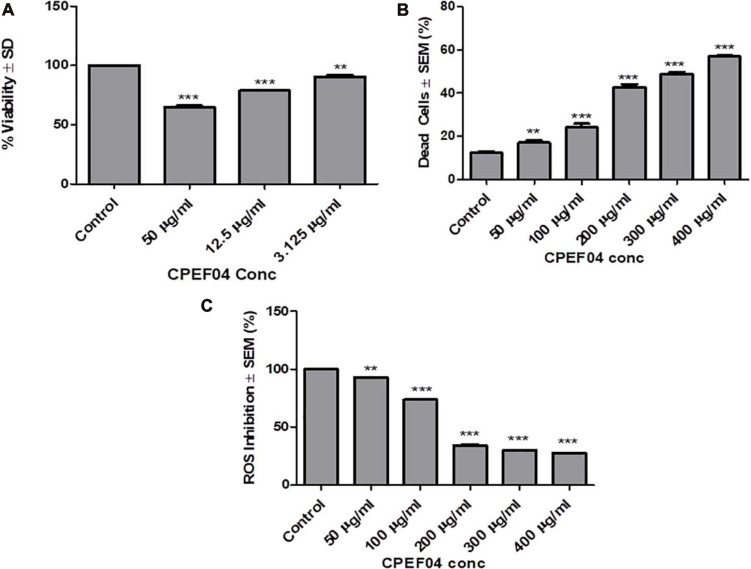
Effect of CPEF04 culture filtrate dried powder (CFDP) on HEK 293T normal cell lines **(A)**, HeLa cancer line **(B)**, and reactive oxygen species inhibition **(C)**. * defines significance level in comparison to control after unpaired *t* test. ***p < 0.0001.

CPEF04 CFDP was tested for their toxic effect on HEK 293T cells by *in vitro* viability test method using alamar blue as described in section “Materials and Methods.” The data obtained was plotted as a bar graph depicting the percent difference between treated and untreated cells to evaluate the level of cellular viability. The results showed that, after treatment with varying concentrations (3.125–50 μg/ml) of CFDP, there was no significant cell death as compared to control cells, as shown in Figure 11A. Similar findings were highlighted by recent work on *Talaromyces australis* which produces an effective pigment for application at a concentration ≥100 μg/ml (Hernández et al., 2019). The minimum cytotoxicity of CPEF04 CFDP on the non-cancerous HEK293 T cells suggests its safe application to food and or pharmaceutical formulations.

### Live/Dead Cell Assay

CPEF04 CFDP-mediated cytotoxicity was measured on HeLa cancer line as model. The cells treated with various concentrations ranging from 50 to 400 μg/ml showed potential growth inhibitory or cytotoxic effects in a concentration-dependent manner. The concentration of CFDP at 300 μg/ml killed nearly 50% of the HeLa cells. There was nearly saturation when the concentration was increased up to 400 μg/ml. The cytotoxic effect of CPEF04 was studied on human embryonic kidney cell line HEK 293T using alamar blue assay, and the cytotoxicity was negligible, verifying its non-toxic effect to non-cancerous cells (Figure 11B). A study on the common food colorants from *Monascus purpureus* supported our findings and that these concentrations were effective and safe (Martinkova, 1999).

### Intracellular Reactive Oxygen Species

Antioxidant compounds have the capability to eradicate or supress free radical generation and propagation using one or a myriad of different mechanisms. The antioxidant potential of CPEF04 CFDP was evaluated on cancerous cell line HeLa. In order to prove the anti-oxidant potential of CPEF04, we used a flow cytometry-based method after staining the cells with DCFH-DA dye. The fluorescence is directly proportional to the amount of ROS generated inside the cells, which is an indicator of early apoptosis (Kumari et al., 2017). The cells treated with various concentrations ranging from 50 to 400 μg/ml showed a potential anti-oxidative effect in a dose-dependent manner. This gave us evidence for the ROS-inhibitory effects of this unique CPEF04 isolate. The ROS inhibition was nearly satiable from a concentration of 200 μg/ml onward. The percent inhibition of ROS was exclusively near to 50% from a concentration of 200 μg/ml onward. Therefore, we can conclude that the strong anti-oxidative nature of CPEF04 might have constrained the generation of ROS inside the cancerous HeLa cells (Figure 11C). Similar results were also reported after treating cancerous cells with resveratrol (Thomas et al., 2016). The pigments obtained from *T. purpureogenus* also exhibited congruent anti-proliferative and anti-oxidative properties (General et al., 2014; Pandit et al., 2018), making them safe for industrial applications.

## Conclusion

This investigation demonstrates the pigment production capacity of endophytic fungi isolated from the healthy roots of a marine plant, *A. marina*. The most promising strain was identified to be *T. assiutensis*. Among *Talaromyces* strains, this article reports the detailed exploration of its pigment-producing capacity, effect of media composition on pigment and biomass yield, tentative identification of the molecules responsible for pigment production, and their pharmacological potential produced by *T. assiutensis* CPEF04. The results obtained from the studies highlighted that media composition plays an important role in pigment production. Moreover, an enhanced capacity to produce extracellular pigments might assist in reducing the huge cost involved in the down-streaming process and thus further enhances the applicability of this strain at a commercial scale. UPLC–MS-based studies revealed the presence of various bioactive and pigment molecules produced by this strain, thus emphasizing its pharmaceutical potential. The antioxidant, antimicrobial, and anticancer properties shown by the CFDP confirms its great potential for food and pharmaceutical usage. However, detailed studies with respect to the toxicity and stability will also be the key aspects in ushering these pigment-imparting molecules for commercial utility.

## Data Availability Statement

The datasets presented in this study can be found in online repositories. The names of the repository/repositories and accession number(s) can be found in the article/Supplementary Material.

## Author Contributions

MG conceptualized and designed the work. RM, RK, and RD performed the experiments. RM and RK carried out the data analysis and manuscript drafting. SD helped in the identification of the fungal strain. MG and CB contributed toward the critical revision and final approval of the manuscript. All authors contributed to the article and approved the submitted version.

## Conflict of Interest

The authors declare that the research was conducted in the absence of any commercial or financial relationships that could be construed as a potential conflict of interest.

## Publisher’s Note

All claims expressed in this article are solely those of the authors and do not necessarily represent those of their affiliated organizations, or those of the publisher, the editors and the reviewers. Any product that may be evaluated in this article, or claim that may be made by its manufacturer, is not guaranteed or endorsed by the publisher.

## Footnotes

1
http://www.geospiza.com/Products/finchtv.html
