# Supplementary material for: Characterization of an Endophytic Strain Talaromyces assiutensis, CPEF04 With Evaluation of Production Medium for Extracellular Red Pigments Having Antimicrobial and Anticancer Properties
Source: Front Microbiol. 2021 Aug 4;12:665702. doi: 10.3389/fmicb.2021.665702 (PMC8371755; doi:10.3389/fmicb.2021.665702)
Supplement: Supplementary file 1 [file Data_Sheet_1.DOCX]

**Characterization of an endophytic strain *Talaromyces assiutensis*, CPEF04 with optimization of production medium for extracellular red pigments having antimicrobial and anticancer properties**

Rahul Chandra Mishra^1^, Rishu Kalra^1^, Rahul Dilawari^2^, Sunil Kr. Deshmukh^1^, Colin J. Barrow^3^ and Mayurika Goel^1^*

^1^TERI-Deakin Nano Biotechnology Centre, The Energy and Resources Institute (TERI), TERI GRAM, Gurgaon, 122001, India

^2^CSIR-Institute of Microbial Technology, Sector-39-A, Chandigarh, 160036, India

^3^Centre for Chemistry and Biotechnology, School of Life and Environmental Sciences, Deakin University, Waurn Ponds, VIC 3220, Australia

* Corresponding author (mayurikagoel@gmail.com; mayurika.goel@teri.res.in)

**Supplementary material**

**Table S1: Different media used for the optimization of fungal anticancer secondary metabolites production**

| **Liquid Media** | **Composition (g/L)** |
| --- | --- |
| Malt Extract Broth (MEB) | Malt extract 20; Glucose 20; Peptone 1 |
| Potato Dextrose Broth (PDB) | Potato infusion 4; Dextrose 20 |
| Sabourard Broth (SDB) | Tryptone 10; Glucose 40 |
| Glucose Yeast Extract Medium (GYEM) | Glucose 10; yeast extract 2 |
| Yeast Extract Peptone Dextrose (YEPD) | Yeast extract 10; Peptone 20; Dextrose 20 |
| Czapek Yeast Extract Dox Broth (CYDB) | NaNO_3_ 2; K_2_HPO_4_ 1; MgSO_4_.7H_2_O 0.5; KCl 0.5; FeSO_4_.7H_2_O 0.01; Sucrose 30, Yeast Extract 5 |
| Defined minimal dextrose (DMD) | (NH_4_)_2_SO_4_ 1; Glucose 30; MgSO_4_.7H_2_O 0.5; K_2_HPO_4_ 1.4; KH_2_PO_4_ 0.6; ZnSO_4_ 0.8; FeSO_4_ 0.8; CuSO_4_ 0.8; NaH_2_PO_4_ 0.8, MnSO_4_ 0.4. |

**Table S2: Biomass and extracellular pigments production by fungal isolate CPEF04 cultivated on seven different growth media.**

| **Media composition** | **Biomass (g/L)** | **Extracellular Pigments (mg eqv. carmine L-1)** |
| --- | --- | --- |
| PDB | 5.16 ± 0.06 | 348.17 ± 2.28 |
| YEPD | 7.01 ± 0.04 | 227.73 ± 3.66 |
| CYDB | 2.91 ± 0.01 | 42.01 ± 2.08 |
| GYEM | 2.11 ± 0.03 | 42.22728 ± 2.85 |
| MEB | 2.77 ± 0.08 | nd |
| SDB | 5.27 ± 0.06 | 59.21744 ±1.14 |
| DMD | 6.36 ± 0.06 | nd |

nd: not detected

**Figure S1: Calibration plot for Carmine standard**

| **Carmine Concentration (mg/L)** | **λ _max_ (494)** |
| --- | --- |
| 5 | 0.052 |
| 10 | 0.054 |
| 25 | 0.22 |
| 50 | 0.472 |
| 100 | 0.796 |
| 200 | 1.58 |
